# The effect of micro-learning on learning and self-efficacy of nursing students: an interventional study

**DOI:** 10.1186/s12909-022-03726-8

**Published:** 2022-09-07

**Authors:** Ladan Zarshenas, Manoosh Mehrabi, Leila karamdar, Mohammad Hasan Keshavarzi, Zahra keshtkaran

**Affiliations:** 1 Community Based Psychiatric Care Research Center, School of Nursing and Midwifery, Shiraz University of Medical Sciences, Shiraz, Iran; 2 Department of E-Learning in Medical Sciences, Virtual School, Center of Excellence for e-learning in Medical Sciences, Shiraz University of Medical Sciences, Shiraz, Iran; 3 Clinical Education Research Center, School of Medicine, Shiraz University of Medical Sciences, Shiraz, Iran; 4 Nursing Department, Community Based Psychiatric Care Research Center, School of Nursing and Midwifery, Shiraz University of Medical Sciences, Shiraz, Iran

**Keywords:** Microlearning, E-learning, Clinical education, Self-efficacy, Multimedia

## Abstract

**Background:**

In the present age, e-learning has been playing a good role in educational and clinical settings along with face-to-face training. This study aimed to determine the effect of distance class using micro-learning contents on learning outcomes and self-efficacy in the clinical education of nursing students in 2021.

**Methods:**

This research is a quasi-experimental pre-test-post-test study conducted at Shiraz University of Medical Sciences. The study population consisted of 46 nursing students who were recruited by the full census method. Students were randomly divided into two groups of intervention and control. Before and after educational interventions in both groups, students' learning and self-efficacy were evaluated using a questionnaire. Data Analysis was done using descriptive and analytical statistical methods and with SPSS software version 23.

**Results:**

The results revealed a statistically significant difference in the mean score of clinical learning level of nursing students between the control and experimental groups after the intervention (*p* = 0.041). Also, the difference between the mean score of self-efficacy in the intervention group before and after the training was statistically significant (*p* = 0.001).

**Conclusion:**

Micro-learning is an effective training method for raising learning outcomes and self-efficacy among nursing students, especially in internship units. This method is recommended since multimedia pays attention to all learning styles of learners and affects the learning outcomes and self-efficacy of learners.

## Background

An effective response to the rapid changes in today's world requires rapid learning and adaptation to new methodologies [1]. Although many teaching methods have been suggested in various literature, the lecture is one of the oldest and, maybe still, the most widely used teaching methods in universities around the world. Due to the limitations of traditional methods such as lecturing, many experts emphasize changing teaching methods [2]. The increasing growths of information technology and electronic devices as well as the limitations of traditional education have led to the introduction of e-learning as an alternative or complementary method in the field of medical education [3]. In general, e-learning refers to the use of electronic systems such as computers, the Internet, multimedia disks, electronic journals, virtual newsletters, etc., which are used to reduce traffic and save time and money while learning would be expedited and easier [4].

Studies have shown that more than 70% of clinical educators use traditional methods, and this is while more than 90% of nursing students prefer to use new methods of evaluation and training over traditional methods [5]. Therefore, choosing the appropriate teaching method in clinical disciplines such as nursing is very important due to the interaction with the patient and can make education very attractive and effective [6]. Using traditional learning methods is time-consuming, costly, and difficult, while in e-learning the content is available and does not require commuting to attend class and the time required for learning is reduced by 25–30%. Also, the attitude of instructors towards using this training method is changing over time. In this regard, Collins' findings showed that teachers have a positive attitude to e-learning as a teaching aid [7]. In microlearning that is related to e-learning, content and training are presented in smaller sections sequentially. The main and complete application of micro-learning is using mobile. Learning includes brief written tutorials, graphic transcripts, podcasts and video clips [8]. Micro-learning is evolving with significant growth and importance among learning management professionals [9] and as an educational strategy has a positive effect on the knowledge and confidence of health professionals in performing procedures, maintaining knowledge, reviewing and learning [10].

Research has shown that nurses with a higher sense of self-efficacy provide better performance and care than those with lower self-efficacy. Understanding self-efficacy as a predictor of nurses' behavior plays an important role in their professional performance [11]. Considering that no direct research has been done on micro-learning and its impact on learning and self-efficacy in clinical education of students in the country, this study aims to investigate the impact of micro-learning as one of the new methods of e-learning on learning and self-efficacy in clinical education of nursing students.

## Methods

### Research design and setting

The present study is an interventional study with a pre-test-post-test design that was performed on 46 nursing students of Shiraz Nursing School who were studying in the second semester of the 2021 academic year and currently undertaking community health internships.

### Sampling

Students were enrolled in the study by census method. They were randomly divided into two groups of intervention (*n* = 21) and control (*n* = 25). The willingness to participate in the study, informed consent, and current participation in the community health internship were among the criteria for entering the study. The exclusion criteria were an unwillingness to continue cooperation.

### Instruments

The data collection tool included a demographic information questionnaire including age, sex, and grade point average. The Scherer Self-Efficacy Questionnaire (Sherer et al., 1982) contains 17 items that are rated on a 5-point scale from strongly disagree (score 1) to strongly agree (score 5). The maximum score is 85 and the minimum is 17 [12]. This questionnaire is used to measure general self-efficacy in social and general skills. Items include four areas: clinical (5 questions), theory (4 questions), motivational (4 questions) and organizational (4 questions). In the study of Karbasi et al., the content validity with experts was used to determine the validity of the questionnaire. To determine the reliability of the self-efficacy questionnaire, a test–retest with a two-week interval was used. The results of Cronbach's alpha coefficient was 0.89, which indicates that its reliability was optimal [13]. A learning questionnaire was prepared based on the topics of vaccination. This questionnaire has 20 four-choice questions that was reviewed and approved by the professors of the Community Health Nursing Department.

### Procedure

After approving the research project and obtaining ethical approval, and coordinating with the officials of the School of Nursing, the students were selected according to the inclusion criteria. Written informed consent was obtained from all students while stating the objectives of the research. In this study, students were included in the study by census method and were randomly divided into two groups of intervention (*n* = 21) and control (*n* = 25).

The e-content in this research was developed in the form of short educational videos. Researcher tried to work based on the principles and standards of multimedia [14] and the first principles of instructional design [15] and was provided to the audience on the WhatsApp social network.

For this purpose we write the first text then we record the sound based on the text. Then we design in PowerPoint software the appropriate slides based on educational principles and we developed the videos and then edited them for final format. We finally developed 5 videos with mean time of 5 min.

**Fig. 1:**
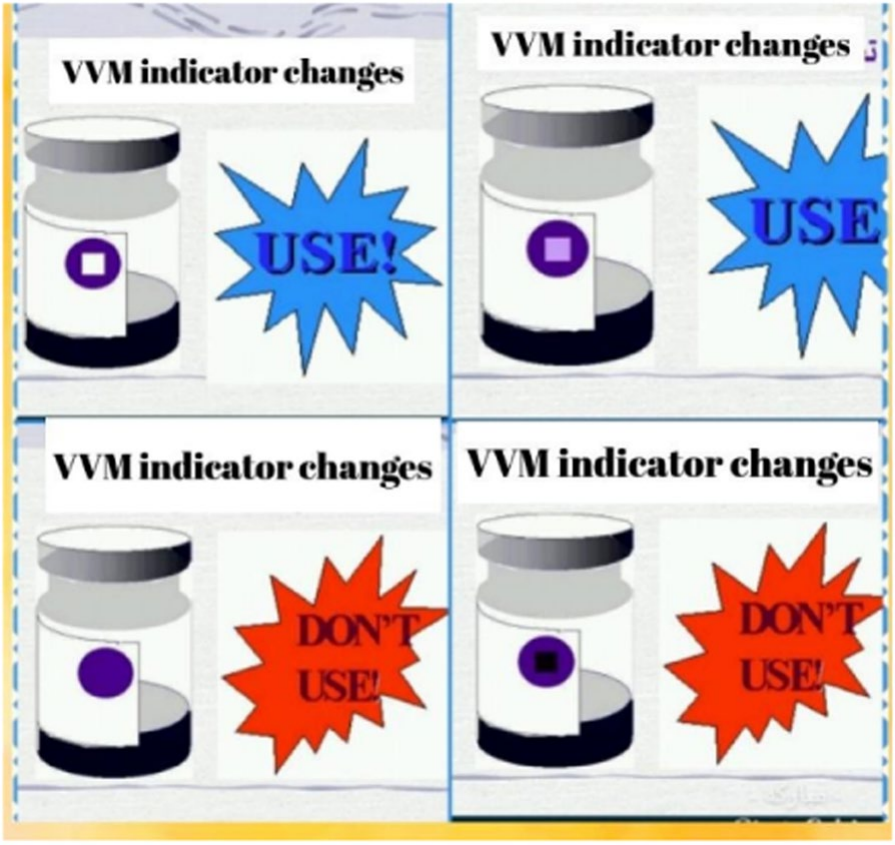
Changes in the vial index of vaccines

**Fig. 2:**
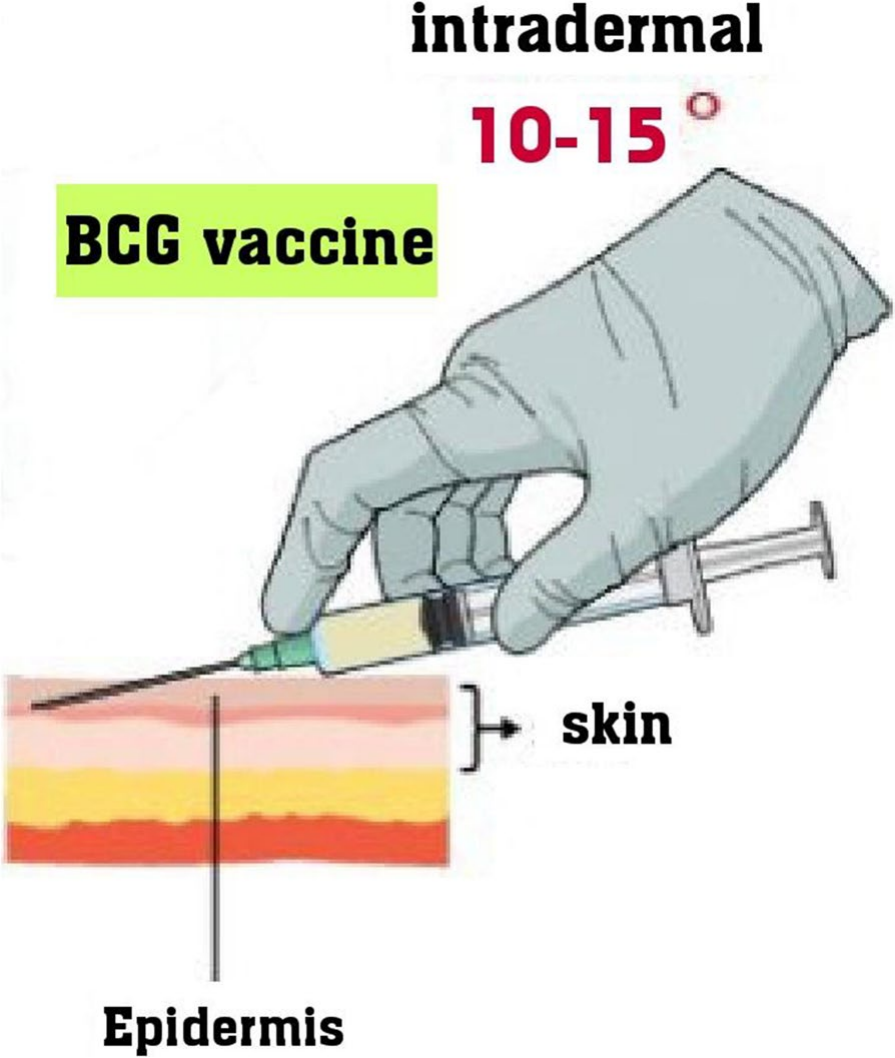
How to inject BCG vaccine

**Fig. 3:**
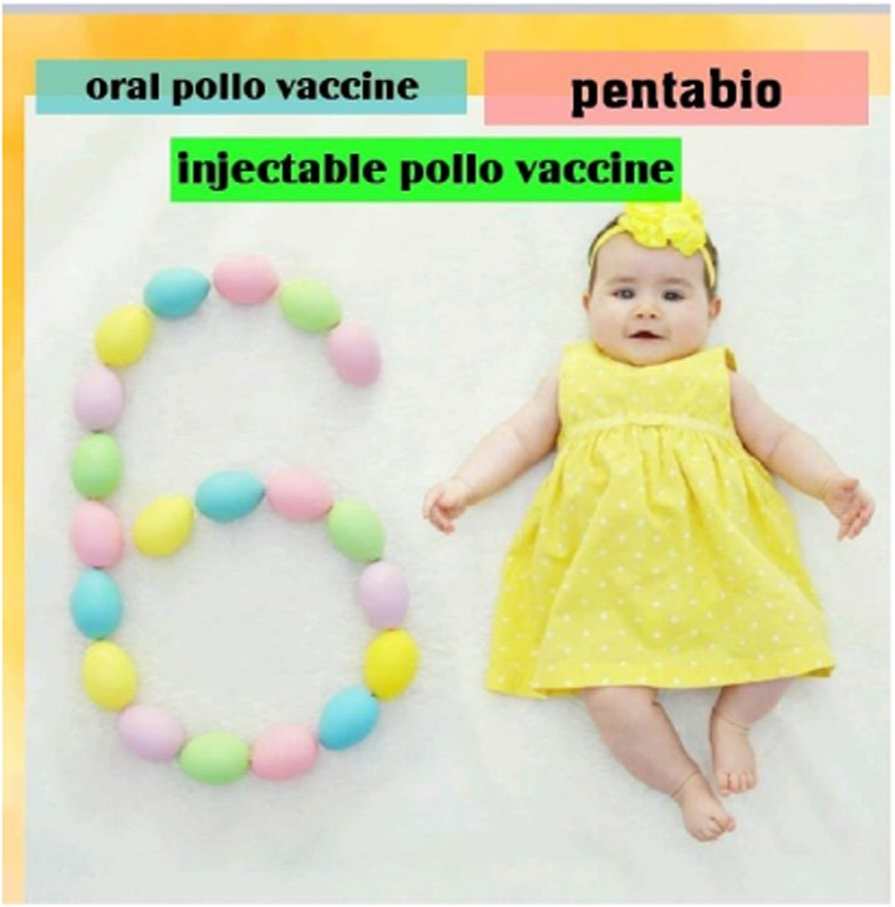
6 month vaccines

The content was about the types of vaccines needed by children, principles of cold chain, contraindications to vaccination, how to properly inject the vaccine, complications of vaccines and child care after vaccination that was provided at the same content in both intervention and control group. Sample images from videos are provided in Figs. 1, 2 and 3.

The objectives of the research were explained to the students of the intervention group and informed consent was obtained. After that, the training in the control group was made based on the educational objectives of routine methods of lectures and questions and answers. In addition to routine training, the students of the intervention group were provided with micro learning content following the educational topics of the course in the form of short videos during the internship through the virtual network. During the training, the researcher communicated with the students through a virtual network and answered their questions. Demographic information questionnaire, Scherer self-efficacy questionnaire, informed consent form and learning assessment questionnaire were completed by students of both groups on the first day of internship. At the end of the internship, the questionnaires were completed again by the two groups.

### Statistical methods

Analysis was performed using descriptive statistics methods (mean and standard deviation) and statistical methods including independent and paired t-test and SPSS software version 23.

## Results

The results showed that 56% of the respondents from the control group and 38.1% of the experimental group were male and 44% of the control and 61.9% of the experimental group were female. The majority of students in the two groups of control (20 students, 83.3%) and experiments (18 students, 85.7%) were under 25 years old.

Kolmogorov–Smirnov test was used to evaluate the data distribution status. Due to the lack of significance of test values, the results of this test showed that the data distribution of research variables in control and intervention groups follows a normal distribution. Therefore, to test the hypotheses of this study, the parametric paired comparison tests and the Student's t-distribution (independent t-test) were used. The learning assessment test in the control group showed a difference in the mean score of clinical learning level of nurses in the control group in the subject of vaccination before and after the training course, which was statistically significant (*p* = 0.001). Also, the results of the Paired Comparison test indicated a difference in the mean score of clinical learning level of students in the experimental group before and after the educational intervention, which was statistically significant according to the level of significance (*p* = 0.001) (see Table 1).The results of the independent t-test showed that the scores between the control and experimental groups before the educational intervention were not significantly different (*p* = 0.973). The results also indicated that the mean score of learning level of control and experimental group students after the educational intervention was statistically significant (*p* = 0.041) (see Table 1).

**Table 1:** Comparison of learning between experimental and control groups before and after the intervention

Variable	Group	Before to intervention	After intervention	*p*-value
		Mean ± SD	Mean ± SD	
Learning	Control	9/08 ± 3/71	12/40 ± 3/07	*P* < 0.001
	Experimental	9/12 ± 3/16	14.29 ± 3/21	*P* < 0.001
Within Group *p*-value		0/973	0/041	
Effect Size		0.655		

Regarding the level of self-efficacy, the results showed no difference in the mean score of self-efficacy level of the control group before and after the training course, which was not statistically significant with the *p*-value of 0.425. The mean score of the self-efficacy level of the experimental group students before and after the intervention was statistically significant (*p* = 0.001) (see Table 2).

**Table 2:** Comparison of the self-efficacy levels before and after intervention in the experimental and the control group

Variable	Group	Before to intervention	After intervention	Between *p*-value
		Mean ± SD	Mean ± SD	
Learning	Control	39/72 ± 7/95	40/12 ± 7/43	0/698
	Experimental	40/62 ± 7/56	44/33 ± 5/94	0/042
Within Group *p*-value		0/425	*P* < 0.001	
Effect Size		0/504		

## Discussion

The results of the self-efficacy test revealed that the traditional training method had no significant effect on nurses 'self-efficacy, while distance class using micro-learning contents had a significant effect on nurses' self-efficacy. In distance class using micro-learning contents, because the educational content is produced and presented in small and capsule sections, the information with small volume is faster and easier to memorize, short and small contents are available for the learner at any time and place. The educational content could be saved easily on devices such as mobile phones, tablets, laptops, etc. and the learner is not required to physically attend the classroom. The findings of the present study are consistent with the study of Khoshnoodifar et al. (2019), which was conducted to determine the level of knowledge, skills and satisfaction of nurses from in-service education of cardiopulmonary resuscitation using two traditional and electronic training methods [16]. Our research also corroborates the previous findings of Pourteimour et al. (2018) which showed the improvement of the attitude and practice of nursing students in preventing drug errors in the pediatric unit after e-learning [17].

The results of data analysis showed that both traditional and distance class using micro-learning contents had a positive effect on the learning outcomes but the comparison of learning levels of nurses revealed that the effect of micro learning on nurses' learning was greater than traditional education. A study by Zarshenas et al. (2016) showed that in both traditional methods and the use of electronic resources and e-learning, multimedia CDs increase learners' knowledge and self-efficacy in the field of osteoporosis [18]. In a favorable correlation with our findings, Nikou et al. confirmed the impact of mobile-based micro-learning and assessment on the learning performance and motivation of high school students [19]. Another study, in good agreement with that of ours, by Nemanahmad et al. (2016) showed that the use of audio podcasts as a micro-learning tool has positive effects on students' learning and reinforces the overall learning outcomes [20].

Since in our study, the contents were presented in the form of video clips, to be more attractive to the audience and also the fact that micro-learning addresses different needs of learners, a positive effect on their learning outcomes and self-efficacy was observed. In his research on medical students in 2021, Sözmen showed that the rate of learning biochemistry in students with the micro learning was higher than the traditional method [21]. In a study by Mohamed et al. conducted on 108 high school students, the results showed that the micro-learning group had 18% better learning outcomes than the traditional education group [22], which confirms the findings of our study. Similar studies indicate the positive effects of the micro-learning on learning outcomes [23] and [24], which lend support to the present research.

Since the purpose of medical education is to improve the health of the individual, society and families, the use of e-learning methods in nursing curricula can help nurses to provide the most scientific, practical and accurate care [25].

### Limitations

It is plausible that the small number of samples in the control and experimental groups, probably due to a low number of admissions, could have influenced the results obtained. COVID-19 related conditions and difficult access to students and professors were other problems since the internship groups attended in smaller groups and to different clinics. We solved the problem of reaching samples by frequent visits of the research team to clinics. Another limitation was the possibility of transferring micro contents from the experimental group to the control group. This led to deviations in the results of the intervention. To solve this problem, the intervention group received training after the control group.

## Conclusions

With the development of electronic technologies and the tendency to digital literacy among learners, we are witnessing its increasing use in various professions, including the academic era. The distance class using micro-learning contents easily engages the learner's mind and memory due to its shortness, attractiveness, and the use of images, animation, and text with sound. The results of this study showed that distance class using micro-learning contents had a positive effect on learning and self-efficacy in clinical education of nursing students and its effect was more than the traditional education method. The distance class using micro-learning contents, which presents educational content in the form of small pieces of text, audio and video, can attract more audience and increase the amount of learning. Therefore, distance class using micro-learning contents is suggested as an effective educational method, especially in nursing training units.

## Data Availability

The datasets during the current study are not publicly available due to confidentiality of the students’ data, but they will be available upon reasonable request.

## Abbreviation

SPSS: Software package used for Statistical Analysis
